# Effect of mandibular first molar mesialization on alveolar bone height: a split mouth study

**DOI:** 10.1186/s40510-019-0275-z

**Published:** 2019-06-10

**Authors:** Nicolas Göllner, Jonas Winkler, Peter Göllner, Nikolaos Gkantidis

**Affiliations:** 1 Private Practice, Spitalgasse 16, CH-3011 Bern, Switzerland; 2 Department of Orthodontics and Dentofacial Orthopedics, University of Bern, Freiburgstrasse 7, CH-3010 Bern, Switzerland

**Keywords:** Alveolar bone loss, Tooth movement, Tooth agenesis, Skeletal anchorage, Molar angulation

## Abstract

**Objectives:**

To evaluate the risk of vertical alveolar bone loss (ABL) in mesialized mandibular permanent molars due to space closure in patients with unilateral second premolar agenesis. The contralateral side served as control.

**Subjects and methods:**

Twenty-five retrospectively selected subjects (median age 14.9, range 12.0, 31.9 years) were analyzed. Space closure (approximately 10 mm) was performed using skeletal anchorage. ABL was measured at mesial and distal sites of first molars in pre- and post-treatment panoramic radiographs. Measurements were corrected for distortion and magnification of radiographs. Molar angulation according to the occlusal plane was also evaluated. Permutational multivariate analysis of covariance (MANCOVA), followed by pairwise comparisons, was performed.

**Results:**

MANCOVA resulted in no difference in ABL between the distal sites of mesialized molars and the control sites. On the contrary, there was statistically higher ABL, at the mesial sites of mesialized versus non-mesialized molars (*p* = 0.042; median 0.19 mm; range − 0.82, 1.33); though the difference was not clinically relevant. In the space closure side, mesially, only two patients had ABL higher that 1 mm. No patient had a severe bone level height defect (> 3 mm distance from the cementoenamel junction) at any point.

When testing differences in molar angulation between sites and from pre- to post-treatment condition, no significant difference was detected (*p* > 0.05, median − 1.9°, range − 13.5, 6.2).

**Limitations:**

This is a retrospective study on panoramic radiographs.

**Conclusions:**

Space closure through extensive tooth movement was identified as a risk factor for vertical ABL, at the mesial sites of mandibular first molars. However, the amount of ABL was not clinically relevant, and thus this treatment option is considered safe in terms of ABL.

## Introduction

Agenesis of permanent teeth is a common dental anomaly with an overall prevalence of 6.4% (95% CI 5.7, 7.2) [1]. The most commonly missing tooth is the mandibular second premolar, which is missing in slightly more than half of the patients with tooth agenesis [2]. Treatment options for this condition involve retention of the primary second molar as long as possible, restoration with a fixed partial denture or a dental implant, counterbalancing extractions in case that other extractions are needed for other reasons in the mandible and orthodontic space closure through mesialization of posterior teeth. Many clinicians consider the latter option as treatment of choice, especially for younger patients, who will undergo orthodontic treatment for other malocclusion issues [3], since the long-term survival rate of the alternative options is questionable [4, 5]. Furthermore, in case of premature loss of the primary second molar, space closure could prevent supra-eruption of the opposing teeth or tipping of the adjacent molar. Additionally, it could possibly facilitate the eruption of third molars. Total treatment costs might also be reduced if orthodontic treatment is planned for other purposes.

However, the orthodontic space closure may also be related to certain adverse effects affecting tooth integrity, such as external apical root resorption (EARR) [3], or periodontal support, such as alveolar bone loss (ABL) [6, 7]. These factors are considered to affect long-term tooth survival in extreme cases and especially when they both affect the same tooth to a large extent [8], thus leading to a detrimental increase of the clinical crown/root ratio.

A recent split-mouth controlled study showed that the risk for EARR is increased in mesialized mandibular permanent molars to the second premolar agenesis site, but the amount of EARR is not clinically relevant [3]. Thus, in terms of EARR, orthodontic space closure is considered a safe option. However, other factors that are crucial for long-term tooth survival, such as the periodontal support, should also be considered when evaluating the outcome of a treatment option. The potential effect of orthodontic treatment on periodontal tissues, including the alveolar bone height, is an important consideration in treatment decision-making, especially in complex cases [6]. The mesialization of mandibular molars toward a narrower part of the jaw, where a tooth was extracted, can be considered such a complex case [6, 9, 10].

Thus, the aim of the present study was to compare the ABL in unilaterally mesialized mandibular molars, using skeletal anchorage, compared with the contralateral teeth of the same patient. The advantage of this split-mouth study is, that it controls for important confounding factors, such as individual predisposition and mouth hygiene, which critically affect periodontal response.

## Materials and methods

### Sample

All patients treated by one experienced orthodontist in a private practice in Bern, Switzerland, between 1998 and 2010 were considered potential for inclusion in the study. Out of a cohort of 105 patients, based on eligibility, 25 patients (16 females, 9 males) who fulfilled the following criteria were assessed in the study.

Inclusion criteria:

- Permanent dentition
- Unilateral agenesis of mandibular second premolar
- Space closure in the agenesis side performed through mesialization of posterior teeth using skeletal anchorage
- Clear pre-treatment and post-treatment panoramic radiographs with no obvious distortions

Exclusion criteria:

- Systemic diseases
- Previous orthodontic treatment
- Sign of spontaneous mesial posterior tooth movement due to exfoliation of the deciduous second molar on the agenesis site
- Signs of generalized periodontal bone loss or other severe periodontal conditions, including severe bone width loss in the agenesis side

All radiographs were obtained by a single operator, using the same machine (Soredex, Cranex 3CEPH). Accurate patient positioning in the radiographic machine was achieved through three positioning lights (midsagittal, Frankfort, focal through). The focal trough light was set at every patient at the level of the canine. The post-treatment radiograph was obtained after the end of the entire active orthodontic treatment.

Patients were selected irrespective of malocclusion and facial pattern. The median patient age at the beginning of treatment was 14.9 years (range 12.0–31.9 years; 3 patients > 18 years), and the median treatment duration was 2.6 years (range 1.1–4.9 years). The treatment duration was calculated from the timepoint of the start of active treatment with full-fixed or segmented orthodontic appliance till the removal of the whole full-fixed appliance.

In 21 patients, the predecessor deciduous tooth on the agenesis side was extracted 2–12 months before placing the skeletal anchorage and starting active space closure. Only in 4 cases the deciduous tooth was already missing at the start of orthodontic treatment. Following initial teeth alignment, space closure was performed on 0.017″ × 0.025″ or 0.019″ × 0.025″ Ni-Ti wires. The posterior teeth of the agenesis side were mesialized using skeletal anchorage and elastic forces, over a space previously occupied by a deciduous second molar, which was expected to be approximately 9.83 ± 0.50 mm [11, 12]. Depending on the overall needs of each case, mini screws (Aarhus Anchorage Screw System) or palatal implants (Straumann SLA 4.2-mm-long and 4.1 mm or 4.8 mm wide, Basel, Switzerland) were used as anchorage units. Palatal implants were placed in patients with agenesis in both jaws (*n* = 7), whereas mini screws were used in patients with agenesis only in the mandible (*n* = 18). In cases of miniscrew placement the mesialization force was exerted by elastomeric power chains, changed by the patients once every 2 weeks, to avoid force decay. In cases with palatal implants, class II intermaxillary elastics were used (1/4″, 4.5 oz., changed every 12 h), with the upper teeth anchored through the palatal implant. The regular recall appointment was every 6–8 weeks. Mini screws were explanted after completion of space closure, whereas palatal implants were kept in place until the end of treatment.

**Fig. 1:**
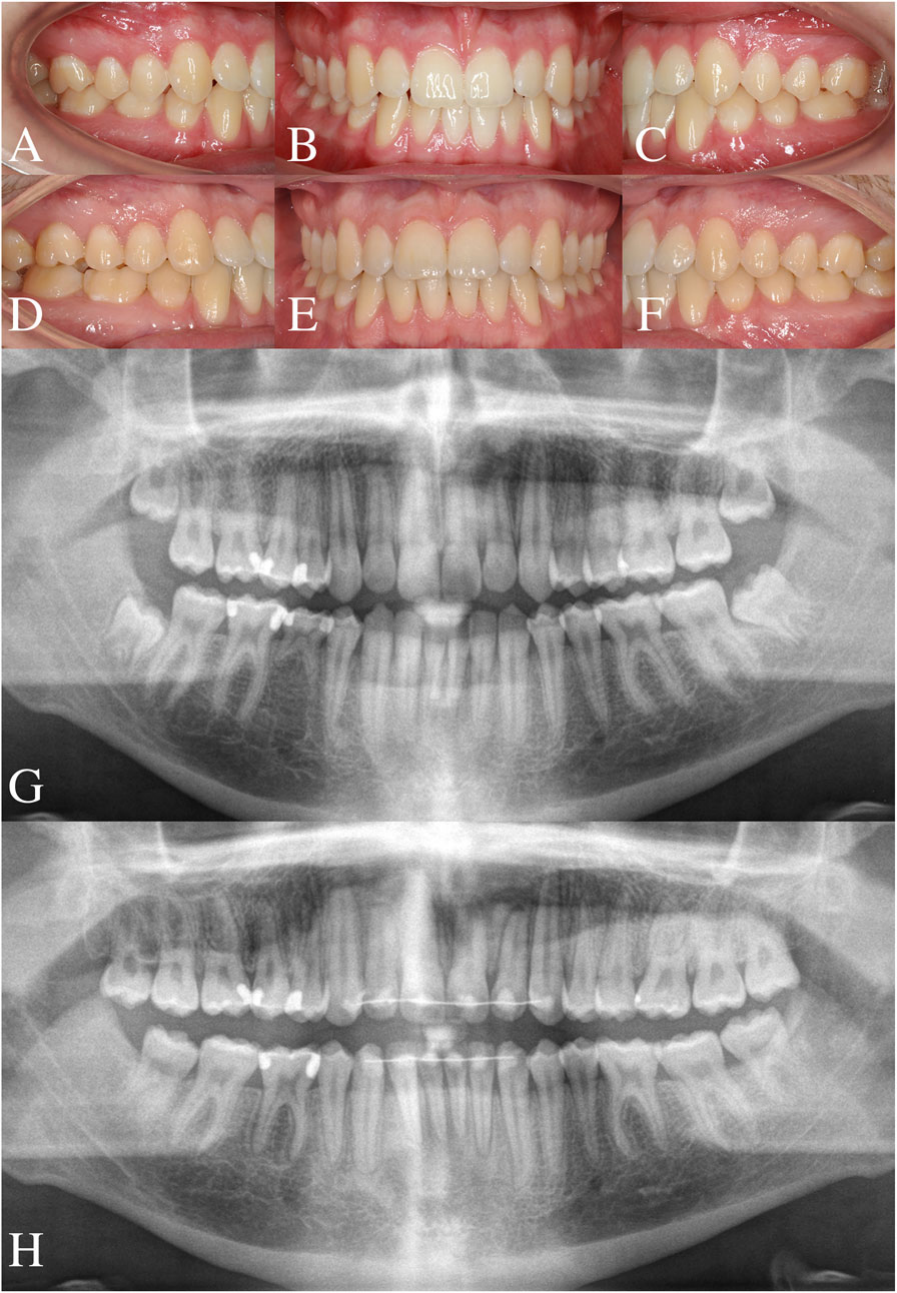
A typical case of unilateral space closure. **a**–**c**, Pretreatment photographs. **d**–**f** Post-treatment photographs. **g**, **h** Pre- and post-treatment panoramic radiograph

All patients had full fixed orthodontic appliances in both arches from second molar to second molar from the start of active treatment or after space closure. Thus, the molars were mesialized using either unilateral segmental fixed appliances in the mandible from first premolar to first molar or full-fixed appliances in both arches from second molar to second molar (Roth prescription, slot size 0.022 in; American Orthodontics, Sheboygan, WI) depending on the clinical condition (Fig. 1).

### ABL assessment

For ABL measurement, all panoramic radiographs were scanned (Perfection V700 scanner; Epson America, Long Beach, CA) with a resolution of 600 dpi at a scale of 1:1 and saved in portable network graphics format. ABL measurements were to be performed on the mesial and distal side of mandibular first molars on both sides of the mandible of each patient. For blinding reasons, the first mandibular permanent molars were cropped from the panoramic radiograph (Paint Software; Microsoft, Redmond, WA) in the mesial side allowing a 2 mm distance mesial to the CEJ point and removing all information that could lead to identification of the presence or type of adjacent tooth. Cropped images were parameterized with random numbers between 0 and 1000 generated on https://www.random.org/. The subsequent images were converted from portable network graphics format to tagged image file format (light image resizer 4 software, version 4.7.3.1; Pixmeo, Bernex, Switzerland) and imported into OsiriX Lite (version 6.5.2; http://www.osirix-viewer.com), which was installed on an iMac 27-in computer (Mac OsX 10.6.8; Apple, Cupertino, CA). The numbers of each radiograph linked with the patient data were saved in a separate file.

**Fig. 2:**
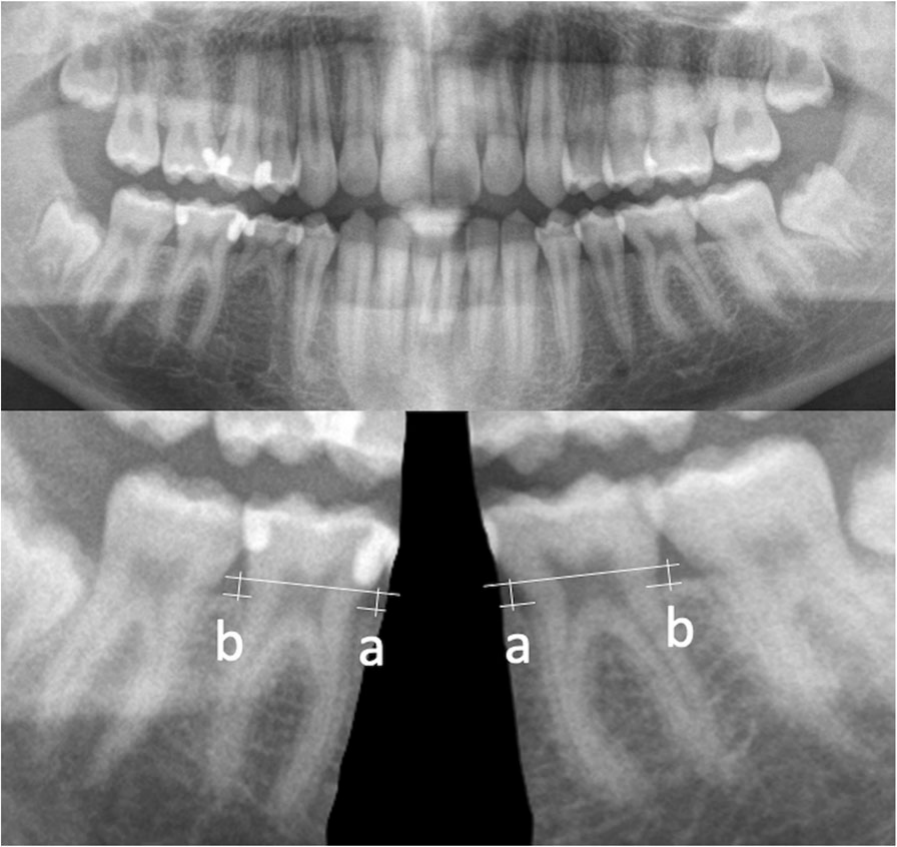
Measurements of alveolar bone loss (ABL) at the mesial (**a**) and distal (**b**) side of the first molar. The lower images are cropped from the upper image to ensure blinding, and they are also magnified to ensure measurement precision

The first two authors performed all measurements independently within a week in a blinded manner, as described below. The tooth images were analyzed on the screen one by one. For better visualization of the tooth structures, the raters were allowed to adjust contrast and intensity and to enlarge the images as needed. Bone level was measured in the pre- and post-treatment panoramic radiographs at mesial and distal sites of the first mandibular permanent molars. The vertical distance of a line connecting the mesial and distal cementoenamel junction (CEJ) points from the most occlusal point of the adjacent bone in each side was defined as bone level measurement (Fig. 2). In cases where the CEJ points were not clearly identifiable in both sides of the tooth, the CEJ line was drawn to be parallel to the occlusal level of this tooth and vertical to its long axis.

Bone level was also measured in cases where the second mandibular permanent molar was not fully erupted (did not reach the occlusal level). This was the case in four agenesis and three control sites.

Bone height measurements on the panoramic radiographs were corrected for magnification and distortion according to the results of a pilot study [3]. For magnification correction, original measurements on the right side were multiplied by 0.714 and on the left side by 0.707. Image distortion was corrected by using the cusp to furcation distance in the pre- and post-treatment radiograph.

### Molar angulation assessment

The second author performed these measurements once and repeated 26 randomly selected measurements for error identification. Blinding was not possible in this case. The angulation of first permanent molars of both agenesis and control sites was measured according to the posterior occlusal level of each corresponding site, without considering the second and third molars. A 90° angle between the long axis of the molar and the posterior occlusal level was considered the starting point. Thus, positive values indicate a mesial angulation and negative values a distal angulation, according to the starting position.

### Statistical analysis

The statistical analysis was carried out by using the SPSS (v.20.0, SPSS Inc., USA), PERMANOVA [13, 14], and PERMDISP [15] software.

Levene’s test revealed homogeneity of variances in all cases. Data were tested for normality through the Shapiro-Wilk test and were not normally distributed in all cases. Thus, non-parametric statistics were applied.

Intra- and inter-examiner agreement on measurements were tested with the Wilcoxon signed-rank test. Random error was assessed through Dahlberg’s formula [16]. In the absence of any systematic error, the mean between the two measurements by the two examiners would be used for further analyses.

Differences in ABL due to treatment or in molar angulation were evaluated using permutational multivariate analysis of covariance (MANCOVA), with a factorial fixed-effects model. In the first case, the continuous dependent variable was the bone level height difference between T0 and T1. Two crossed factors and their possible interaction were analyzed: mesiodistal location (fixed factor; 2 positions: mesial or distal) and side membership (fixed factor; 2 sides: space closure or control). Regarding molar angulation, this was the continuous dependent variable. Two crossed factors and their possible interaction were analyzed: treatment status (fixed factor; 2 statuses: pre or post-treatment) and side membership (fixed factor; 2 sides: space closure or control). Patient was set as a covariate in both cases to account for possible matching and clustering effects. Pair-wise a posteriori comparisons were performed when significant differences were detected by the multivariate model [17, 18].

Permutational analysis of multivariate dispersions (PERMDISP) was used to determine whether potential differences between any pair of groups were due to location, dispersion, or a combination of the above.

In all cases, a two-sided significance test was carried out at an alpha level of 0.05.

## Results

No systematic error was detected between the ABL measurements by the two examiners, and thus the average measurements were used for further analysis. The mean random error of measurements between the two examiners was 0.23 mm (range 0.19, 0.32). A negligible systematic error was evident between the repeated angulation measurements (mean absolute difference 1.08°; range: 0.01, 2.80; *p* = 0.004, Wilcoxon signed rank test), and thus it was not further investigated.

When testing the ABL from pre- to post-treatment condition, the side membership factor approached the level of statistical significance (d.f. = 1, *F* = 2.95, *p* = 0.085) (Table 1). Permutational analysis of multivariate dispersions showed that differences between space closure and control side were not due to dispersion (d.f. = 1, *F* = 0.01, *p* = 0.993), and thus they were due to location. Pair-wise a posteriori tests between side membership factor, within the levels of mesiodistal location factor, showed that there were significant differences in ABL at the mesial sites (*p* = 0.042), whereas no difference was evident at the distal sites (Table 1).

**Table 1:** Non-parametric MANCOVA on ABL (difference between pre- and post-treatment levels) at different mesiodistal locations (mesial or distal), and side memberships (space closure or control)

	d.f.	*F*	*p*
Covariate (patient)	1	0.18	0.677
Side	1	2.95	0.085*
Mesiodistal location	1	2.10	0.156
Side × mesiodistal location	1	0.95	0.339
Residual	95		
Total	99		
Comparison^1^	p		
Mesial space closure vs. mesial control	0.042**		
Distal space closure vs. distal control	0.639		

Two crossed factors and their interactions were analyzed in each case having “patient” as a covariate: mesiodistal location (fixed factor; 2 locations) and side membership (fixed factor; 2 sides)

*Close to statistical significance level, *p* < 0.05

^1^Pair-wise a posteriori tests between side membership factor, within the levels of mesiodistal location factor

**p < 0.05; Wilcoxon signed-rank test: *p* = 0.015

Bone level mesially to first molars, at the space closure side, had a median ABL of 0.19 mm compared to control side (range − 0.82, 1.33) (Table 2). In the space closure side, mesially, only two patients had ABL higher that 1 mm; namely, 1.16 and 1.47 mm. Distally, the higher ABL observed was 0.93 mm. In the control site, the higher ABL observed was 0.80 mm mesially and 0.43 mm distally. No patient in the sample had a severe bone level height defect at any point. Bone height distance from CEJ was in all cases less than 3 mm in all pre- and post-treatment tests.

**Table 2:** Alveolar bone height measurements (mm). Descriptive statistics for each study parameter

Dependent variable	Side		Range	
		Median	Min	Max
Mesial bone height (T0)	Agenesis	0.83	0.07	2.68
	Control	0.81	0.50	2.72
	Difference	− 0.01	− 1.64	0.84
Mesial bone height (T1)	Agenesis	1.01	0.41	2.47
	Control	0.85	0.22	1.46
	Difference	0.13	− 0.76	1.10
Distal bone height (T0)	Agenesis	0.77	0.05	1.98
	Control	0.87	0.50	2.80
	Difference	− 0.11	− 0.85	0.62
Distal bone height (T1)	Agenesis	0.83	0.21	2.17
	Control	0.80	0.20	1.45
	Difference	− 0.02	− 0.77	0.73
Mesial bone height (T1–T0)	Agenesis	0.15	− 0.69	1.47
	Control	− 0.04	− 1.49	0.80
	Difference	0.19	− 0.82	1.33
Distal bone height (T1–T0)	Agenesis	− 0.08	−1.09	0.93
	Control	0.08	− 2.29	0.43
	Difference	0.00	− 0.71	1.33

*T0* pre-treatment; *T1* post-treatment

When testing differences in molar angulation between sides and from pre- to post-treatment condition, no significant difference was detected (*p* > 0.05, Table 3). Before treatment, the first permanent molars were mesially angulated by a median of 14.0° (range 7.1, 27.6) in the agenesis and by a median of 14.7° (range 8.0, 29.8) in the control side. Treatment led to a slight uprighting by a median of − 1.9° (range − 12.8, 9.9) in the agenesis and − 1.9° (range − 13.5, 6.2) in the control side as well.

**Table 3:** Non-parametric MANCOVA on molar angulation at different treatment statuses (pre- or post-treatment), and side memberships (space closure or control)

	d.f.	*F*	*p*
Covariate (patient)	1	0.01	0.935
Side	1	0.23	0.634
Treatment status	1	1.78	0.183
Side × treatment status	1	0.85	0.365
Residual	95		
Total	99		

Two crossed factors and their interactions were analyzed in each case having “patient” as a covariate: treatment status (fixed factor; 2 statuses) and side membership (fixed factor; 2 sides)

## Discussion

In the present study, we tested the effect of the mesialization of a molar toward a premolar space on alveolar bone height and evaluated the changes in tooth angulation. To our knowledge, this is the first study that tested this. Furthermore, the split-mouth design allowed for control of important confounding factors, such as individual predisposition, skeletal pattern, treatment conditions, or mouth hygiene.

Results showed significant alveolar bone loss (ABL) from pre- to post-treatment condition at the mesial sites of the mesialized molars, compared to contralateral control sites, whereas no difference was evident at the distal sites. However, the additional ABL on the mesial side, compared to the control side, was minimal (median 0.19 mm; range − 0.82, 1.33) and not clinically relevant. The maximum ABL detected in the sample was 1.5 mm. In a previous split-mouth study on the same group of patients, space closure through mesialization of the mandibular first molar, due to agenesis of the second premolar, was considered to be a valid and safe treatment option also in terms of risk for EARR [3].

The present results are in agreement with previous studies, which tested the mesialization of mandibular second and third molars on a first molar site and showed favorable outcomes [7, 19]. However, our results are not directly comparable with these for various reasons. They were both uncontrolled, they included primarily adult patients, part of the edentulous space was spontaneously closed, and they did not correct measurements for distortion and magnification. Finally, in our study, first molars were mesialized to a premolar site, thus to a narrower alveolar bone site. Our results are also in agreement with those of a controlled study on first premolar extraction orthodontic patients [20], although still not directly comparable.

The fact that there was minimal ABL in the mesial sites of space closure cases compared to controls, but there was no difference between the two groups in the distal sites, suggests that this bone loss might not be attributed solely to tooth movement. Probably the absence of a functional dental unit in the temporarily edentulous space or the movement of a molar toward a narrower alveolar bone site is the cause of this minimal ABL and not the extensive tooth movement. Unfortunately, no previous study, which showed similar results for the space closure site, measured also distal sites [7, 19].

In the current sample, no difference was detected in molar angulation between space closure and control sites, as well as from pre- to post-treatment. This was expected, since, according to the eligibility criteria, no patient with any sign of spontaneous closure of the agenesis space was included in the study.

The present study showed a median treatment duration of 2.6 years (range 1.1–4.9 years). This corresponds with the treatment time reported in the literature for extraction cases [21], since extractions tend to increase treatment time compared to non-extraction cases [21]. This is also the case for space closure in patients with mandibular premolar agenesis. However, in unilateral agenesis cases, segmented fixed appliances can reduce patient discomfort during the space closure phase. Nevertheless, patients should be informed accordingly prior to treatment start.

The present study suggests that space closure through the mesialization of permanent molars toward a premolar space is a valid treatment option that does not jeopardize the periodontium, although wide teeth are moved toward a place where narrower teeth were supposed to be present. Since the ABL risk proved to be minimal, as was also the case for EARR [3], and when considering the suboptimal long-term results of the alternative options [4, 5], orthodontic space closure might be the treatment of choice in such patients, especially in younger ages.

### Limitations

Although a carry-across effect is always a potential limitation in split mouth studies [22], a significant systemic effect that could contaminate the results of one side over the other is not expected in the present study for the main outcome tested, since space closure was performed using skeletal anchorage.

The fact that different skeletal anchorage means were used in this patient group might had caused different responses of the bone, due the different biomechanical approaches. However, any potential effect would not have been clinically relevant in this study, since bone height defects attributed to molar mesialization were minimal, in all cases tested. Furthermore, the primary strength of the study, which is the split-mouth design, accounted for this potential confounding factor.

The limitations of this study can be attributed mainly to its retrospective nature. Retrospective data collection might introduce selection and detection bias. To reduce selection bias, all patients who fulfilled the inclusion criteria were included in the study. To control for detection bias, strict blinding measures were applied and ABL measurements were performed by two examiners independently.

Another limitation could be that ABL was evaluated at panoramic radiographs, which are the routine radiographs performed in an orthodontic practice at present, although measurements were corrected for distortion and magnification. Several studies indicated a more valid measurement of ABL on periapical radiographs, but there is considerable agreement with those on panoramic radiographs [23, 24]. Both these radiographs have the limitation of presenting a three dimensional structure in two dimensions. Thus, the alveolar bone width, which might be considered an important factor for the periodontal response when the space closure option is adopted, was not evaluated in the present study due to the 2D nature of the available data. Ideally, high-resolution cone-beam computed tomography scans would be preferable, but these were not justified in the present study population due to radiation protection issues [25]. Finally, potential changes in soft-tissues, such as gingival recessions, or long-term treatment effects could not be evaluated in this study. Thus, for a thorough evaluation of this condition, studies including clinical periodontal examination and intraoral scans before and after treatment would be required to evaluate hard and soft tissues in a three-dimensional manner.

The angulation measurement on panoramic radiographs is another limitation [26], although certain studies support this method if the patient positioning is proper [27]. In any case, the split-mouth design of our study accounts for these and other potential confounding factors.

## Conclusions

The findings of the present split-mouth study that controlled for additional causative factors, suggest that mesial movement of first molars for second premolar space closure is indeed a risk factor for ABL. However, the observed amount of ABL, measured as bone height reduction, was minimal and not clinically relevant. Thus, this treatment can be considered safe in terms of ABL risk, especially when applied in young patients with mandibular permanent premolar agenesis.

However, to generalize these findings, other factors should also be considered during treatment planning, such as treatment alternatives, age, treatment duration, costs, risk for recessions, or other adverse effects related to alveolar bone width, orthodontic relapse, and occlusal aspects.

## Abbreviations

ABL: Alveolar bone loss
CEJ: Cementoenamel junction
EARR: External apical root resorption
